# Effectiveness of oral clonidine and gabapentin on peripheral neuropathy in diabetic patients in southwestern Iran: a randomized clinical trial

**DOI:** 10.1186/s12902-023-01486-0

**Published:** 2023-10-16

**Authors:** Sajad Hassanzadeh, Soraya Bagheri, Seyed Majid Ahmadi, Seyed Ahmadreza Ahmadi, Isaac Moradishibany, Hosein Dolatkhah, Sajjad Reisi

**Affiliations:** 1 Department of Internal Medicine, School of Medicine, Yasuj University of Medical Sciences, Yasuj, Iran; 2 School of Medicine, Jundishahpour University of Medical Sciences, Ahvaz, Iran; 3 Genetic and Environmental Adventures Research Center, School of Abarkouh Paramedicine, Shahid Sadoughi University of Medical Sciences, Yazd, Iran

**Keywords:** Clonidine, Gabapentin, Peripheral neuropathy, Diabetes, Neuropathic pain, Severity of neuropathic pain

## Abstract

**Background:**

Peripheral neuropathy is not only the most prevalent consequence of diabetes but also the main reason for foot ulceration, disability, and amputation. Therefore, the current study aims to determine the effectiveness of oral clonidine and gabapentin on peripheral neuropathy in diabetic patients.

**Methods:**

This 12-week, randomized, and parallel-group trial was conducted to compare the efficacy of oral clonidine and gabapentin with gabapentin alone in diabetic patients in southwest Iran during the first half of 2021. Thirty patients with type 2 diabetes with peripheral neuropathy as assessed by a visual analog scale (VAS) and divided into two groups of 15 patients, treated for up to three months. The data were analyzed using SPSS-21 software. In order to report the results, descriptive indices, independent t-test, one-way analysis of covariance (ANCOVA) and analysis of variance with repeated measures were used.

**Results:**

The mean and standard deviation of the age of the participants in the clonidine + gabapentin group was equal to 50.20 ± 7.44, and in the gabapentin group was equal to 50.47 ± 7.57 (t = 0.10, P-value = 0.923). This research showed a significant difference between the clonidine + gabapentin group and with gabapentin group in terms of neuropathic pain and the severity of neuropathic pain (P < 0.001).

**Conclusions:**

According to this research results, clonidine + gabapentin can reduce neuropathic pain and the severity of neuropathic pain in diabetic patients. Therefore, it is recommended that healthcare professionals with diabetes expertise prescribe these medications to reduce neuropathic pain and its severity.

**Trial registration:**

This study was registered in the Iranian Clinical Trials System with the ID (IRCT20211106052983N1) on 14/01/2022.

## Background

Diabetes Mellitus (DM) is a metabolic disorder caused by low insulin production or function, resulting in chronic hyperglycemia [1]. This illness has been considered one of the leading health problems affecting more than 400 million individuals across the globe [2]. The prevalence of this disorder is predicted to be 10.2% and 10.9% by the end of the years 2030 and 2045, respectively. In addition, the general prevalence of DM is higher in urban areas (10.8%) compared to the countryside (7.2%) [3, 4]. Moreover, according to the national reports of diabetes prevalence, 9.2 million people are predicted to suffer from diabetes by 2030 [5]. Due to sedentary lifestyles and unhealthy food, most diabetes (especially type 2) is on the rise [6, 7]. The main consequences of DM include neuropathy, nephropathy, and retinopathy [8].

As a significant cause of foot ulceration, disability, and amputation, Diabetic Peripheral Neuropathy (DPN) is the most prevalent complication resulting from diabetes. More than 50% of diabetic patients undergo Diabetic Neuropathy (DN), which affects their nervous system [9]. Furthermore, 20–30% of individuals diagnosed with DPN experience severe neuropathic pain, usually intense, chronic, and difficult to manage or treat [10, 11]. In addition, one-third of patients with DPN also suffer from symptoms such as tingling (pins and needles), increased sensitivity to heat and coldness, numbness, and loss of sensation in feet [12]. The pain caused by neuropathy can negatively affect patients’ quality of life and dramatically increase treatment costs. In addition to these costs, annual therapeutic payments are twice higher for individuals with peripheral neuropathy [13].

Currently, there are few treatments for DN, most of which include many side effects. In previous investigations, the results of melatonin [14, 15], caffeine [16], capsaicin [17], vitamin B_12_ [18], alpha-lipoic acid [19], vitamin E [20], acupuncture [21], gabapentin and duloxetine [22, 23], pregabalin and gabapentin [24], gabapentin [25, 26], the topical combination of clonidine and pentoxifylline [27], and clonidine gel with capsaicin cream has been studied [28]. Anticonvulsants are common medications for treating peripheral neuropathy but are not sufficiently compelling and include side effects [29]. Gabapentin is an anticonvulsant used for DPN treatment [30]. On the other hand, in addition to their side effects, the effectiveness of pain relievers (oral solution) like pregabalin and duloxetine is varied [31]. Therefore, considering the high degree of DPN and the limited known treatments for managing this illness, it is essential to use medications with acceptable efficiency and effectiveness [32]. According to the results of previous studies, clonidine is likely to alleviate neuropathic pain when used topically on the painful area [33, 34]. Clonidine is an alpha-2-adrenergic agonist with sympatholytic effects [35]. Alpha-2 adrenergic receptors are available on epidermal pain receptors [36]. The sources of neural signals causing pain in DPN are unknown [37], and neither are the effect mechanisms of oral clonidine + gabapentin on the functions of the peripheral nervous system in diabetic patients. Therefore, the current investigation aims to determine the effectiveness of oral clonidine and gabapentin on peripheral neuropathy in diabetic patients.

## Methods

The current research is a randomized clinical trial on two groups with a pre-intervention phase and three post-intervention assessments (after two, four, and eight weeks). The population consisted of all diabetic patients with peripheral neuropathy who sought medication in the diabetes clinic of Yasuj during the first half of 2021. The inclusion criteria were: (1) Informed consent of all participants, (2) being diagnosed with diabetes and having symptoms of peripheral neuropathy, (3) being aged between 30 and 60, (4) being able to participate in the study, (5) not suffering from chronic (cardiovascular, kidney disease, and hyperthyroidism or hypothyroidism), neurological, and severe psychiatric illnesses, (6) not being pregnant or involved in breast-feeding, (7) no simultaneous use of medicines (antipsychotics, analgesics, or tricyclic antidepressants) which can affect neuropathic pain, and (8) not having hypotension (having + 11 blood pressure). Also, our exclusion criteria were (1) being highly allergic to clonidine, (2) being diagnosed with neuropathy for causes other than diabetes, (3) experiencing worsened symptoms due to taking the medicines, and (4) having a severe drop in blood pressure or drug interaction.

### Procedure

In the present study, 49 diabetic patients were included using a purposeful sampling method. Thirty-four people entered the research process based on the entry criteria. Still, in operation, in each of the research groups, according to the exit criteria, two people left the research process. Finally, analyzed the information from 30 participants. They were then assigned to the clonidine + gabapentin and gabapentin groups randomly (according to the random allocation law). 15 participants were assigned to each group. In the present study, the researcher and the participants did not have any information about the drugs received. The researcher had only assigned the participants to the groups and evaluated them in the evaluation stages, but he had no information about the drugs that the participants were taking. In addition, the participants had no knowledge about which group they were in. The participants had no information about the number and type of medication prescribed for the opposite group. However, the attending physician was aware of the type of prescription drugs for each group based on the inclusion criteria and control of their side effects (drugs). Therefore, since the researcher and the participants did not know about the treatment process, the current research was double-blind (for the researcher and the participants). The participants in the experimental group received a daily clonidine dosage of 0.10 milligrams (Clonidine tablets were used at night) and a gabapentin capsule (100 milligrams) for eight weeks. However, the individuals in the control group took only gabapentin capsules (100 milligrams) daily for eight weeks. The participants in both groups completed the demographic—background information forms and the study measurements at all stages of the investigation.

### Measurements

#### Michigan Diabetic Neuropathy Symptom score (DNS)

DNS combines scores from a neurological examination and standard nerve conduction stimulation. A 128-hertz diapason generates vibration to calculate this variable, and pain is induced on the back of the examinee’s toe using a sharp tool. During this procedure, the examinee is asked yes/no questions and is expected to answer. Responses with seven or more correct answers and one to seven or no correct answers are interpreted as average, weak, or lack of sensation, respectively. In addition, muscular strength is also examined in the distal muscles and rated on ranges of 0-normal, 1-weak-medium, and 2-very weak. Score 3 is given when examinee’s are unable to contract their muscles deliberately. The current tendon reflexes requiring facilitation and having no reflexes are scored by 0, 1, and 2, providing a maximum total score of 46. Higher scores than six are associated with neuropathic pain [38].

#### The pain visual analog scale (VAS)

In the current investigation, the patients were asked to rate their pain on the visual analog scale (VAS). This measurement consists of a scoring system varying between 0 (no pain) to 10 (unbearable pain), being known as the pain ruler [39].

### Statistical analysis

In the current research, frequency, percentage, mean and standard deviation indicators were used to examine descriptive results. Also, to check the homogeneity of demographic and background variables, the Chi-square test, Fisher’s exact test, and independent t-test were used. In addition, an independent t-test was used to investigate the differences between groups of neuropathic pain variables and neuropathic pain severity in different stages of the research. Finally, in order to check the results of research hypotheses, univariate analysis of covariance (ANCOVA) and variance test with repeated measurements were used.

## Results

Table 1 provides information on the descriptive indexes and the results of homogeneity of gender, and medications of the participants according to their groups.

**Table 1:** The descriptive indexes result of homogeneity gender, and medications of the participants according to their groups

Variable		Clonidine + Gabapentin		Gabapentin		Homogeneity	
		Frequency	Percentage	Frequency	Percentage	Statistic	P-value
**Gender**	Men	4	26.7	7	46.7	1.29	0.450*
	Women	11	73.3	8	53.3		
**Medication**	Unknown	8	53.3	8	53.3	0.82	1**
	B Calcium	2	13.3	1	6.7		
	Angiotensin inhibition	4	26.7	5	33.3		
	B angiotensin and angiotensin inhibitory	1	6.7	1	6.7		

* Chi-squared test; ** Fisher’s exact test

As can be seen in Table 1, the groups do not difference significantly in terms of gender, and medication (P > 0.05). The mean and standard deviation of the age of the participants in the clonidine + gabapentin group was equal to 50.20 ± 7.44, and in the gabapentin group was equal to 50.47 ± 7.57, and based on the independent t-test results, it was shown that there was no significant difference between the research groups in terms of age (t = 0.10, P-value = 0.923). Also, the mean and standard deviation of the duration of type 2 diabetes (months) for the participants in the clonidine + gabapentin group is equal to 139.66 ± 95.80 and for the participants in the gabapentin group is equal to 154.80 ± 63.59, and based on the independent t-test results, there was no significant difference between them (t = 0.51, P-value = 0.614). In addition, the mean and standard deviation of the duration of diabetic peripheral neuropathy (months) for the participants in the clonidine + gabapentin group is equal to 46.80 ± 45.59 and for the participants in the gabapentin group. equal to 24.13 ± 17.39 and based on the independent t-test results, there was no significant difference between them (t=-1.80, P-value = 0.083). Table 2 presents information on the descriptive indexes and background information homogeneity of the patients suffering from neuropathic pain in the pre-intervention and post-intervention stages (two, four, and eight weeks after the initiation of the intervention).

**Table 2:** The descriptive indexes and background information homogeneity of the patients suffering from neuropathic pain in pre-intervention and post-intervention stages (two, four, and eight weeks after the initiation of the intervention)

Assessment stages	Variable	Group Response	Clonidine + Gabapentin		Gabapentin		Homogeneity	
			Fre	Per	Fre	Per	Statistic	P-value
pre-intervention	Symptoms of diabetic neuropathy in the form of pain	Yes	14	93.3	14	93.3	0	1^*^
		No	1	6.7	1	6.7		
	Symptoms of diabetic neuropathy in the form of tingling	Yes	15	100	15	100	N/A	N/A
		No	N/A	N/A	N/A	N/A		
	Comorbidity to heart, kidney and mental diseases	Yes	N/A	N/A	N/A	N/A	N/A	N/A
		No	15	100	15	100		
	Medications used to treat diabetes	Ins	5	33.3	6	40	2.61	0.281^**^
		Med	7	46.7	3	20		
		1via2	3	20	6	40		
	Medications used to treat the neuropathic complication of diabetes	No	15	100	15	100	N/A	N/A
		Gabapentin	N/A	N/A	N/A	N/A		
	Blood pressure history	Yes	7	46.7	7	46.7	0	1^*^
		No	8	53.3	8	53.3		
	Complaints about the quality of sexual activity	Yes	13	86.7	7	46.7	6.39	0.035^**^
		No	1	6.7	7	46.7		
		No spouse	1	6.7	1	6.7		
	Diapason	1024	N/A	N/A	2	13.3	2.14	0.483^*^
		512	15	100	13	86.7		
Two weeks after the start of the intervention	Symptoms of diabetic neuropathy in the form of pain	Yes	3	20	14	93.3	21.85	< 0.001^**^
		No	N/A	N/A	1	6.7		
		Improved	12	80	N/A	N/A		
	Symptoms of diabetic neuropathy in the form of tingling	Yes	3	20	15	100	20	< 0.001^*^
		Improved	12	80	N/A	N/A		
	Complaints about the quality of sexual activity	Yes	5	33.3	7	46.7	13.53	0.002^**^
		No	1	6.7	7	46.7		
		Improved	8	53.3	N/A	N/A		
		No spouse	1	6.7	1	6.7		
Four weeks after the start of the intervention	Symptoms of diabetic neuropathy in the form of pain	Yes	N/A	N/A	8	53.3	13.29	< 0.001^**^
		No	N/A	N/A	1	6.7		
		Improved	15	100	6	40		
	Symptoms of diabetic neuropathy in the form of tingling	Yes	N/A	N/A	8	53.3	18.48	< 0.001^**^
		No	9	60	N/A	N/A		
		Improved	6	40	7	46.7		
	Complaints about the quality of sexual activity	Yes	1	6.7	2	13.3	7.86	0.018^**^
		No	1	6.7	7	46.7		
		Improved	12	80	5	33.3		
		No spouse	1	6.7	1	6.7		
Eight weeks after the start of the intervention	Symptoms of diabetic neuropathy in the form of pain	Yes	N/A	N/A	7	46.7	9.46	0.006^**^
		No	2	13.3	1	6.7		
		Improved	13	86.7	7	46.7		
	Symptoms of diabetic neuropathy in the form of tingling	Yes	N/A	N/A	8	53.3	21.74	< 0.001^**^
		No	11	73.3	N/A	N/A		
		Improved	4	26.7	7	46.7		
	Complaints about the quality of sexual activity	Yes	N/A	N/A	3	20	11.97	0.003^**^
		No	1	6.7	7	46.7		
		Improved	13	86.7	4	26.7		
		No spouse	1	6.7	1	6.7		

NOTE: Fre = Frequency; Per = Percentage; * Chi-squared test; ** Fisher’s exact test

As shown in Table 2, based on the results of Fisher’s exact test, there is a significant difference between the research groups in terms of complaints about the quality of sexual activity in the first stage of evaluation (P < 0.05). Also, in the second stage of evaluation based on the results of Fisher’s exact test in terms of symptoms of diabetic neuropathy in the form of pain and complaints about the quality of sexual activity (P < 0.005) and based on the result of Chi-square test in terms of symptoms of diabetic neuropathy in the form of tingling (P < 0.001), there was a significant difference between the research groups. Also, in the third and fourth stages of evaluation based on the results of Fisher’s exact test, there is a significant difference between the research groups in terms of symptoms of diabetic neuropathy in the form of pain, symptoms of diabetic neuropathy in the form of tingling, and complaints about the quality of sexual activity (P < 0.05). In Table 3, descriptive indices and independent t-test results for neuropathic pain (based on the DNS scores) and neuropathic pain intensity (based on the visual analog pain scale scores) of the participants based on the stages evaluation and their groups are shown.

**Table 3:** The Descriptive indices and independent t-test results for neuropathic pain and neuropathic pain intensity of the participants based on the stages evaluation and their groups

Variable	Assessment stages	Clonidine + Gabapentin group		Gabapentin group		Leven’s Test		independent t-test	
		M	SD	M	SD	F	P-value	t	P-value
**Neuropathic pain**	First	23.53	2.29	19.87	5.04	7.12	0.013^*^	2.56	0.019^*^
	Second	15.80	3.30	19.33	4.82	1.31	0.261	-2.34	0.026^*^
	Third	10.47	2.36	16.33	4.83	3.98	0.056	-4.22	< 0.001^**^
	Fourth	8.13	2.36	15.53	4.73	3.08	0.090	-5.42	< 0.001^**^
**Pain severity**	First	9.07	1.62	9.33	1.17	2.97	0.096	-0.51	0.610
	Second	6.33	1.68	8.53	1.60	0.54	0.468	-3.68	0.001
	Third	4.73	1.16	7.73	1.49	0.11	0.742	-6.16	< 0.001^**^
	Fourth	4.40	0.63	7.20	1.47	5.59	0.025^*^	-6.76	< 0.001^**^

* P < 0.05; ** P < 0.01

Note: M = Mean; SD = Standard deviation

As shown in Table 3, based on the independent t-test results, there is a significant difference between the research groups in terms of neuropathic pain in the first stage of evaluation (P < 0.05). In this regard, the ANCOVA test was used to control the effect of the pre-test to check the results of neuropathic pain in the second, third, and fourth stages of evaluation. But, according to the independent t-test results, there is no significant difference between the study groups in the initial evaluation regarding pain severity (P > 0.05). In this regard, a variance test with repeated measurements was used to check the results of pain severity.

Table 4 presents ANCOVA results for comparing neuropathic pain between the study groups in the second, third, and fourth assessment stages.

**Table 4:** The ANCOVA results for comparing the neuropathic pain between the study groups in the second, third, and fourth assessment stages

Index stage	Source	SS	df	MS	F	P-value	Partial Eta Squared
Second assessment	Pre-test	338.48	1	338.48	65.63	< 0.001	0.71
	Group	279.91	1	279.91	54.27	< 0.001	0.67
	Error	139.26	27	5.16			
	Total	9829	30				
Third assessment	Pre-test	125.26	1	125.26	12.09	0.002	0.31
	Group	373.99	1	373.99	36.09	< 0.001	0.57
	Error	279.81	27	10.36			
	Total	6050	30				
Fourth assessment	Pre-test	116.83	1	116.83	11.49	0.002	0.30
	Group	526.74	1	526.74	51.78	< 0.001	0.66
	Error	274.63	27	10.17			
	Total	5003	30				

* Effect Size: A numerical value that determines the difference between data and a statistical hypothesis

It is demonstrated in Table 4 that according to the results of ANCOVA, there are significant differences between the groups in terms of neuropathic pain in the second (F = 54.27, P < 0.001), third (F = 36.09, P < 0.001) and fourth (F = 51.78, P < 0.001) stages of assessment. Furthermore, according to eta squared effect sizes, prescribing clonidine + gabapentin (compared to a single prescription of gabapentin) during stages two, three, and four of assessment can explain 0.67, 0.57, and 0.66 of the between-group variances in neuropathic pain. Table 5 provides the multivariate test results for the severity of neuropathic pain (based on the pain visual analog scale).

**Table 5:** The results of multivariate test for severity of neuropathic pain

Source	Test	SS	df	MS	F	P-value	Partial Eta Squared
Assessment stage	Greenhouse-Geisser	208.33	2.184	95.38	102.04	< 0.001	0.78
Assessment stage × Group	Greenhouse-Geisser	35	2.184	16.02	17.14	< 0.001	0.38

As shown in Table 5, the effects of the assessment stages on the multivariate model of neuropathic pain severity have been significant, meaning that the severity of neuropathic pain has had significant variations over time (P < 0.001, F = 102.04). Moreover, considering the eta-squared effect sizes, the evaluation stages can explain 0.78 of the variances in neuropathic pain severity. Furthermore, the interactive effects of assessment stages s and groups have been significant on the multivariate model of neuropathic pain severity (P < 0.001, F = 17.14). According to the eta squared size effects, the interactive results of evaluation assessments and the groups can explain 0.38 of the neuropathic pain severity variances. Thus, to examine the within-group points of difference in neuropathic pain, the interactive effects of the assessment stages s and study groups are inspected separately. Table 6 details each research group’s results of multivariate test effects for neuropathic pain severity (based on *the pain visual analog scale scores*).

**Table 6:** The Results of multivariate test effects for neuropathic pain severity (based on the pain visual analog scale scores) for each research group

Source	Test	SS	df	MS	F	P-value	Partial Eta Squared
Clonidine + Gabapentin group	Greenhouse-Geisser	204.13	1.830	111.54	74.49	< 0.001	0.84
Gabapentin group	Sphericity Assumed	39.20	3	13.07	29.19	< 0.001	0.68

According to Table 6, there is a significant difference between the assessment stages for the experimental group (clonidine + gabapentin) in terms of neuropathic pain severity (P < 0.001, F = 74.49). A combined prescription of clonidine and gabapentin within the stages can significantly affect the severity of neuropathic pain. Moreover, considering the eta squared value, the drug clonidine + gabapentin can explain 0.84 of the within-group variances in the neuropathic pain severity. Furthermore, as can be seen in Table 6, there are significant differences among the assessment stages for the control group (gabapentin) in terms of neuropathic pain severity (P < 0.001, F = 29.19). Eta squared indicates that gabapentin consumption could explain 0.68 of the within-group variances in the seriousness of neuropathic pain. For this reason, the Bonferroni posthoc test was performed to examine the differences between the assessment stages s in neuropathic pain severity in study groups.

**Fig. 1:**
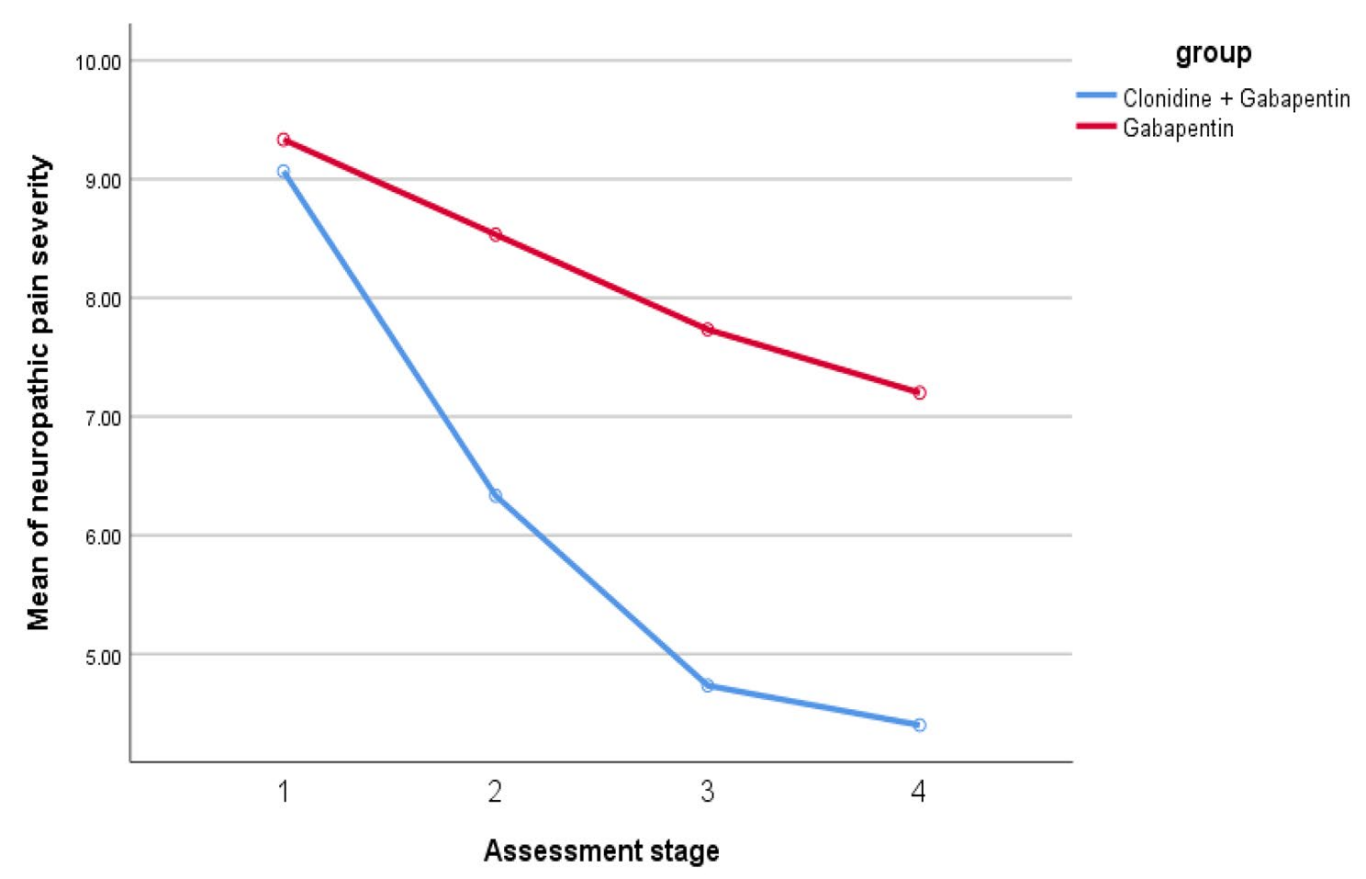
The interactive effects of the assessment stages and the study groups in terms of neuropathic pain severity

According to the results of the Bonferroni posthoc test, in the experimental group (clonidine + gabapentin), there are significant differences in terms of neuropathic pain severity between the first assessment and the other three assessment stages (P < 0.001). Also, the second assessment stages significantly differ from the third and fourth stages in neuropathic pain severity (P < 0.005). However, stages three and four are not significantly different (P > 0.05). Also, based on the results of the Bonferroni test in the control group (gabapentin), a significant difference was shown between the first assessment and the rest of the assessment stages (P < 0.05) and between the second assessment and the third and fourth assessment (P < 0.05). Despite these findings, no significant difference was discovered between stages three and four (P > 0.05). Figure 1 presents the interactive effects of the assessment stages and the study groups regarding neuropathic pain severity.

In addition, based on the results of multivariate effects between groups, there is a significant difference between the research groups regarding the severity of neuropathic pain (F = 22.42, P < 0.001). In addition, eta squared demonstrates that compared to gabapentin, prescribing clonidine combined with gabapentin can explain 0.44 of the between-group variances of the severity of neuropathic pain.

## Discussion

The current research aimed to determine the effectiveness of oral clonidine and gabapentin on peripheral neuropathy in diabetic patients. The results indicated that combining clonidine and gabapentin significantly affected peripheral neuropathy more than a single gabapentin consumption. To the best of our knowledge, to date, no previous investigation has examined the effectiveness of a combined treatment of gabapentin and clonidine (oral solution) on peripheral neuropathy among individuals who have diabetes. Below is information from other studies with similar results. Fulas et al. [27] described a topical combination of clonidine and pentoxifylline that significantly affects neuropathic pain. A research article by Majumdar et al. [40] revealed that gabapentin and clonidine have similar soothing effects before surgeries. Hence clonidine is more effective on laryngoscopy compared to gabapentin. According to a study by Kiani et al. [28], clonidine gel operates more effectively than capsaicin cream in treating DPN-driven pain. Campbell et al. [34] demonstrated that clonidine gel could reduce pain in feet to a considerable extent in patients with painful diabetic neuropathy. Moreover, Chakraborty et al. [41] found that an additional dosage of clonidine to bupivacaine can dramatically extend the effectiveness of anesthetics. According to the points above, our findings are in line with and comparable to these studies. Improving blood sugar control using insulin and antidiabetic medication (oral solution) are proven strategies for decreasing the severity of neuropathy. In this regard, serotonin–norepinephrine-reuptake inhibitors (SNRIs), anticonvulsants, tricyclic antidepressants, and opioid substances are widely used for pain control [11, 42]. Gabapentin is an anticonvulsant initially used as a muscle relaxant and antispasmodic [43, 44]. In addition, gabapentin is utilized for therapeutic purposes in many illnesses, including neuropathy [45]. gabapentin (oral solution) is the first-line treatment for diseases with chronic pain [46]. Gabapentin can affect the positive and negative symptoms of neuropathy [47]. The effect mechanisms of gabapentin on alpha-2 adrenergic receptors [48], which can reduce central sensitivity and facilitate analgesic effects by decreasing the release of stimulated neurotransmitters like glutamate, have been demonstrated in older investigations [49]. Moreover, gabapentin is associated with Ca and Na channels, moderation of monoamine neurotransmitters, and NMDA currents [50]. Therefore, emphasizing the inhabitation of calcium channels, the anticonvulsant activity, and the analgesic effects of gabapentin for neuropathic pain could be explained. However, Chang CY et al. (2014) indicated that gabapentin could not be metabolized in the human body. They also reported that the most common side effects caused by gabapentin (independent of the dosage) are dizziness and sleepiness [51]. In addition, the other side effects of gabapentin include tremors, blurred vision, anxiety, and memory problems [52]. In the present research project, the combination of gabapentin and clonidine was examined. Clonidine facilitates long-term pain reduction by processing an analgesic effect on the spine, but limited studies have focused on it [53].

Clonidine is a high blood pressure medication that can affect the alpha-adrenergic receptors and imidazoline as an agonist [54]. A critical theory about the effect mechanisms of clonidine in pain management describes that many pain signals are generated in the dorsal horn of the spinal cord and then sent to the central nervous system. In this respect, norepinephrine is released from descending inhibit spinal neurons. For this reason, clonidine, which targets alpha-2 adrenergic receptors, could affect pain transmission [55, 56]. According to the findings of previous studies, it has been indicated that clonidine could contribute to reductions in the density of catecholamines dosage. The suppressing effects of clonidine can facilitate the release of catecholamines and better glucose regulation. Moreover, it has been indicated that clonidine and its derivatives are sedative even in lower dosages and can soar blood glucose levels. Therefore, prescribing clonidine for oral solution or injection can raise blood sugar, indicating alterations in the central or peripheral effect mechanisms [57]. Thus, clonidine contains analgesic effects [58] and can play a significant role in diabetic neuropathy pain management [59]. According to the abovementioned points, gabapentin and clonidine have analgesic effects. Both can affect alpha-2 adrenergic receptors.

There were limitations in the present study, which include: not having a group that was prescribed only clonidine. In this regard, it is suggested to consider a group for clonidine alone in future research. Another limitation of the research was the use of a purposeful sampling method, which led to a reduction in the generalizability of the results. In this regard, it is suggested to use a random sampling method to select participants in future research.

## Conclusions

The current research determines the effectiveness of oral clonidine and gabapentin on peripheral neuropathy in diabetic patients. Therefore, according to the results, it has been concluded that a combination of oral clonidine and gabapentin can effectively reduce peripheral neuropathy symptoms in diabetic patients. Consequently, it is suggested that diabetes clinics, hospitals, and specialists prescribe a combination of these drugs for reducing peripheral neuropathy pain in diabetic patients if needed.

## Data Availability

It is possible to access the data after coordination with the corresponding author by email.

## Abbreviations

VAS: Visual analog scale
ANCOVA: One-way analysis of covariance
DM: Diabetes Mellitus
DPN: Diabetic Peripheral Neuropathy
DN: Diabetic Neuropathy
DNS: Michigan Diabetic Neuropathy Symptom Score
SNRIs: Serotonin–Norepinephrine Reuptake Inhibitors
